# *Experimental Physiology* Special Issue: New approaches for old diseases

**DOI:** 10.1113/EP094254

**Published:** 2026-11-05

**Authors:** Daniel H. Craighead, Ronan M. G. Berg, Kristen L. Nowak

**Affiliations:** ^1^ School of Kinesiology University of Minnesota Minneapolis Minnesota USA; ^2^ Centre for Physical Activity Research Copenhagen University Hospital – Rigshospitalet Copenhagen Denmark; ^3^ Department of Clinical Physiology and Nuclear Medicine Copenhagen University Hospital – Rigshospitalet Copenhagen Denmark; ^4^ Department of Clinical Medicine Faculty of Health and Medical Sciences University of Copenhagen Copenhagen Denmark; ^5^ Neurovascular Research Laboratory Faculty of Life Sciences and Education University of South Wales Pontypridd UK; ^6^ Division of Renal Diseases and Hypertension University of Colorado Anschutz Medical Campus Aurora Colorado USA

Chronic ageing‐related cardiometabolic conditions such as hypertension, diabetes mellitus and obesity have long been a significant public health concern and the focus of significant medical and physiological research. Despite having improved our understanding of these conditions and establishing numerous effective preventive measures, chronic disease risk, morbidity and mortality remain high (Hacker, 2024). Therefore, there is an urgent need for further investigation into chronic ageing‐related cardiometabolic diseases to improve public health. The Special Issue on ‘New approaches for old diseases’ called on investigators to submit work that either provided a novel physiological understanding of chronic ageing‐related health conditions or explored a new strategy for preventing or treating chronic conditions. The response to this call was excellent, with a total of 15 papers spanning topics from diabetes mellitus, to cardiovascular health, to kidney function, and more.

Cardiovascular diseases remain the leading cause of death worldwide (Global Burden of Cardiovascular Diseases and Risks 2023 Collaborators, 2025), with ageing being the strongest risk factor (Ferris et al., 2026). Therefore, it is not surprising that many of the contributions to this issue focused on the cardiovascular system. Cardiovascular disease risk accelerates in women during the menopause transition, which is in part attributable to reductions in endothelial function. Endothelial dysfunction and the resulting increased risk for cardiovascular diseases has traditionally been attributed to the loss of oestrogen during menopause. In their review, Nuckols et al. (2026) highlight the changes in other hormones, including follicle stimulating hormone, luteinizing hormone, progesterone and testosterone, that also accompany ageing and menopause and present evidence that these hormones, along with oestrogen, contribute to changes in cardiovascular health. The authors also cover the likely mechanistic pathways involved and emphasize that significant further research on this topic is required.

Stokes et al. (2026) also explored the implications of menopause for vascular function. They specifically sought to examine potential mechanisms underlying the association between the vasomotor symptoms, or hot flushes, and cardiovascular disease risk in postmenopausal women. The authors recruited two groups of postmenopausal females, those experiencing three or more hot flushes per day and those with no history of hot flushes; blood pressure and microvascular endothelial function were assessed in both groups. While there was no difference between groups in blood pressure, those experiencing vasomotor symptoms had a lower reactive hyperaemia index measured via pulse amplitude tonometry. This finding suggests that postmenopausal females experiencing regular hot flushes have impaired microvascular endothelial function, which may contribute to the increased cardiovascular disease risk in this group.

A review by Ferguson et al. (2026) covered vascular contributions to late‐onset Alzheimer's disease, drawing evidence from both clinical and preclinical models. The authors discuss how detrimental changes in both the cerebrovasculature and extracranial arteries contribute to Alzheimer's disease risk, while also assessing potential mechanisms of action, such as oxidative stress and inflammation. Similarly, Cullen et al. (2026) used a mouse model to investigate how elastin insufficiency may contribute to cerebrovascular dysfunction. Middle‐aged elastin haploinsufficient mice had a lower resting cerebral blood flow relative to wild‐type mice but also had a greater cerebrovascular responsiveness to hypercapnia. This finding suggests that elastin insufficiency alone does not impair cerebrovascular reactivity.

Jacob and colleagues (2026) contributed a review on how sex may impact the vascular responses to hypoxia. While the authors highlight that further sex‐specific investigation is required, they make a compelling argument that young women exhibit a vascular protective effect against hypoxia‐induced increases in sympathetic nervous system activity relative to young men.

Atrial fibrillation was a topic of two reviews. First, the review by Thakur and Kalman (2026) highlighted the need for risk factor management as a core component of atrial fibrillation care with ageing. The review by Grigoriou et al. (2026) similarly highlighted the importance of lifestyle modification and risk factor management with atrial fibrillation, specifically in conjunction with catheter ablation treatment. Collectively, these two reviews emphasize the necessity of better incorporation of lifestyle modification into the treatment of atrial fibrillation.

To round out the investigations on cardiovascular function, Mehdizadeh and colleagues (2026) examined how clearing p16‐positive cardiac cells, that is, cardiac cells that are potentially senescent, impacted cardiac function in young versus aged mice. Among the positive effects observed, clearing p16‐positive cells improved cardiac structure and diastolic function in aged mice. This suggests that removal of senescent cells may improve cardiac structure and function with ageing.

Diabetes mellitus is another leading cause of morbidity and mortality, impacting over 500 million people worldwide (GBD 2021 Diabetes Collaborators, 2023). Accordingly, multiple submissions sought to cover both type 1 and type 2 diabetes in the context of ageing and disease risk. Kok et al. (2026) contributed a review covering the gut microbiome as a driver and therapeutic target for treating type 1 diabetes. The authors highlight promising studies where shifts in the gut microbiome are associated with beneficial changes in glycaemic markers; however, they also note the significant heterogeneity among current investigations and call for more rigorous clinical trials on this topic to be performed.

Jafari, Mardani et al. (2026) authored a systematic review and meta‐analysis on the potential benefits of okra (*Abelmoschus esculentus*) for treating diabetes. The meta‐analysis, which included data from 14 clinical trials of patients with different forms of diabetes, indicated that okra supplementation could improve both glycaemic and lipid profiles. However, the authors warn that the results should be interpreted cautiously, as the methodological quality of the included studies was low to very low for most outcomes.

Finally, regarding diabetes mellitus, Harrison et al. (2026) used a type 2 diabetes rat model to investigate the relation between inflammation, the exercise pressor reflex, and mechanical allodynia, that is, where usually innocuous stimuli elicit a pain response. California Davis‐type 2 diabetes rats exhibited mechanical allodynia relative to control (Sprague–Dawley) rats. Furthermore, the onset of allodynia was accompanied by an exaggerated exercise pressor reflex and inflammation, as indicated by elevated C‐reactive protein. This foundational work will aid future investigation to establish if mechanical allodynia can be used to predict cardiovascular events in patients with type 2 diabetes.

The remaining submissions to this special issue covered various topics relevant to other chronic diseases. Koehler et al. (2026) contributed a review on dietary interventions for treating acute kidney injury. This excellent review combines preclinical and clinical data to evaluate approaches such as caloric restriction, periodic fasting, ketogenic diets, pharmacotherapies, and more for treating acute kidney injury.

McGowan and colleagues (2026) contributed a review on lifestyle management for polyendocrine metabolic ovarian syndrome. This review is unique in that it not only covers what is known regarding lifestyle interventions for improving physiological function, it also covers implementation and barriers to meeting lifestyle management strategies in women with polyendocrine metabolic ovarian syndrome. The review concludes with a call‐to‐action for more high‐quality implementation studies.

The final two studies in this special issue examined liver cirrhosis and epilepsy, two chronic health conditions with significant public health impact. Jafari, Mahdavi‐Mazdeh et al. (2026) examined the relation between dietary intake and mortality in an exploratory, prospective cohort study of 121 patients diagnosed with cirrhosis in the previous 6 months. Higher total fat and saturated fat intake were associated with greater risk of mortality during the 60‐month follow‐up period. The authors position these results as hypothesis‐generating and call for subsequent properly powered prospective cohort studies and randomized clinical trials on this topic. Abbasi and colleagues (2026) contributed a cross‐sectional analysis of dietary amino acid intake patterns and odds of having epilepsy. Utilizing the Prospective Epidemiologic Research Studies in Iran Sabzevar Cohort Study, the authors found that intake of predominantly plant‐based amino acids was associated with lower odds of epilepsy, while there was no association between animal amino acids and odds of epilepsy. While cause and effect cannot be determined, this study does advance our understanding of dietary influences on epilepsy.

Collectively, these contributions also highlight the importance of moving beyond conventional disease‐specific approaches towards a more integrated understanding of the physiological mechanisms that underpin ageing and chronic disease. Vascular dysfunction, metabolic dysregulation, inflammation and lifestyle‐related factors frequently intersect, offering opportunities to identify shared therapeutic targets across seemingly disparate conditions. The challenge now is to translate these mechanistic insights into effective, personalised interventions that account for biological diversity, multimorbidity and real‐world implementation. Bridging the gap between physiological discovery and sustainable clinical benefit will be central to developing genuinely new approaches to old diseases.

Overall, this Special Issue illustrates the breadth and depth of the work going on in the physiology community concerning ageing‐related chronic conditions. A large focus was on cardiovascular diseases and diabetes mellitus, two highly prevalent chronic conditions that are leading causes of mortality, while important contributions related to other chronic conditions were made as well. Overall, while significant strides have been made to understand the mechanisms underlying chronic diseases, and in developing effective therapeutic approaches, continued investigation on this topic is required. We hope the work highlighted in this Special Issue can inspire translation into new important research topics. We encourage the physiology community to continue to explore new approaches to prevent and treat ageing‐related chronic diseases to improve the health of all people.

## AUTHOR CONTRIBUTIONS

Daniel H. Craighead: first draft. All authors: conception and design of the work, revising it critically for important intellectual content. All authors approved the final version of the manuscript and agree to be accountable for all aspects of the work in ensuring that questions related to the accuracy or integrity of any part of the work are appropriately investigated and resolved. All persons designated as authors qualify for authorship, and all those who qualify for authorship are listed.

## CONFLICT OF INTEREST

Daniel H. Craighead is part of the *Experimental Physiology* Future Leaders Scheme. Ronan M. G. Berg is Deputy Editor‐in‐Chief of *Experimental Physiology*.

## FUNDING INFORMATION

None.

## GENERATIVE AI STATEMENT

No generative AI tools were used in the preparation of this manuscript.
